# Carboxylated Poly(vinylidene fluoride) Copolymer: A Facile Route to Improve Ultrafiltration Membrane Properties for Aqueous Filtration

**DOI:** 10.3390/membranes16040121

**Published:** 2026-03-30

**Authors:** Yani Jiang, Zihao Zhao, Xianbo Yu, Quangang Cheng, Shaoyu Zou, Yang Zeng, Qiang Huang, Ziran Zhu, Weiwei Zhu, Liping Zhu, Baoku Zhu

**Affiliations:** 1 China-Portugal Belt and Road Joint Laboratory on Advanced Materials, Department of Polymer Science and Engineering, ERC of Membrane and Water Treatment (MOE), Zhejiang University, Hangzhou 310027, Chinazhzhao@zju.edu.cn (Z.Z.);; 2 Zhejiang Research Institute of Chemical Industry Co., Sinochem Lantian Fluoro Materials Co., Ltd., Hangzhou 310023, China

**Keywords:** ultrafiltration membrane, aqueous filtration, poly(vinylidene fluoride) copolymer, carboxyl, antifouling

## Abstract

Poly(vinylidene fluoride) (PVDF)-based ultrafiltration membranes play key roles in aqueous separation fields. However, the inherent hydrophobicity of PVDF always generates higher water permeation resistance and a greater fouling tendency in the filtration process. Different to the widely reported and widely used blending methods of increasing the hydrophilicity of PVDF membranes, the mass-produced hydrophilic PVDF copolymer is expected to be more efficient in producing high performance membranes. For this purpose, the present research offers a new and scalable approach to improving the hydrophilic properties of PVDF-based membranes through amphiphilic copolymers. Using 2-trifluoromethylacrylic acid (MAF) and hexafluoropropylene (HFP), carboxylated PVDF (PVHM) was synthesized following simple radical suspension copolymerization. Via a non-solvent-induced phase separation (NIPS) method, PVHM membranes were prepared and characterized. It was found that the PVHM membranes had enhanced hydrophilicity, permeability, fouling resistance, and alkali resistance compared with PVDF membranes. For the PVHM containing 8.3 wt% MAF, its membrane demonstrated superior static/dynamic fouling resistance to sodium alginate (FRR up to 99.1% for SA). Therefore, carboxylated PVDF polymers show potential for use in the industrial production of high-performance ultrafiltration membranes.

## 1. Introduction

Ultrafiltration (UF) membranes have been widely applied in many water-related fields, such as wastewater treatment, food processing, drug and bio separation, and purification, due to their advantages of low energy consumption, mild separation conditions, simple operation, and high separation efficiency [1,2,3,4]. Among existing polymers, poly(vinylidene fluoride) (PVDF) has been extensively used in microfiltration and ultrafiltration membranes due to its excellent chemical and thermal stability and high mechanical strength [5,6]. When used in aqueous filtration, however, the inherent hydrophobicity of PVDF generates two key disadvantages, namely, higher water permeation resistance and fouling tendency [7].

The hydrophilization of membranes is one of the most common options for improving the permeability and anti-fouling performance and prolonging the service life of membranes for application in the aqueous filtration field [8]. Commonly used modification methods include coating [9,10,11], blending [12,13,14], surface grafting [15], and direct membrane preparation with amphiphilic copolymers [16,17]. Comparing these methods, direct membrane formation from amphiphilic copolymers can overcome the shortcomings of other methods, such as membrane pore blockage, detachment of hydrophilic modifiers from the surface of membrane matrix or internal precipitation, damage to membrane structure, poor component compatibility, and uneven modification [18,19,20,21]. Therefore, the synthesis of amphiphilic copolymers and membrane fabrication are increasing being seen as more advantageous methods for the practical application of UF membranes.

According to previous reports, amphiphilic copolymers, such as sulfonated polyethersulfone [22], sulfonated polysulfone [23,24], sulfonated polyphenylsulfone [25], and carboxylated PVC [26,27], have been produced industrially, while only a few studies have addressed amphiphilic grafted PVDF [28,29,30] and block PVDF [31]. Furthermore, studies on the preparation of carboxylated PVDF via one-step polymerization are scarce. Therefore, this research has significant novelty and practical importance in the industrialization of amphiphilic PVDF.

Amphiphilic PVDF usually combines hydrophilic components and hydrophobic membrane matrix materials by grafting, blocking, and random copolymerization. During membrane formation, hydrophilic components are segregated to the membrane surface to improve the hydrophilicity of polymeric membranes. Among these components, random copolymers are easily synthesized, capable of large-scale production, and have industrial application prospects. It has been reported that directly casting from amphiphilic random copolymers can efficiently improve the permeability, anti-fouling, and antibacterial properties of polymeric membranes [32]. However, most reported amphiphilic PVDF polymers have been synthesized in laboratory conditions via grafting using PVDF as a starting polymer. Their low synthesis efficiency and high cost has limited their industrial-scale production, and so far, the need for high performance PVDF-based membranes has not been met.

To overcome these limitations, carboxyl-containing amphiphilic PVDF copolymers were proposed and synthesized following a radical polymerization process, which can be used for large-scale production. After a series of investigations, it was found that the 2-trifluoromethylacrylic acid (MAF) monomer could easily be copolymerized with the vinylidene fluoride (VDF) and hexafluoropropylene (HFP) monomers to obtain amphiphilic copolymers of P(VDF-*co*-HFP-*co*-MAF) (abbreviated as PVHM) with high yield, molecular weight, and carboxyl content. Investigations into membrane preparation and membrane properties revealed the effectiveness of synthesized carboxylated PVDF in improving membrane performance.

## 2. Materials and Methods

### 2.1. Materials

Commercially available PVDF (Solef^®^6010) was obtained from Solvay Specialty Polymers (Brussels, Belgium). Hydroxypropyl methyl cellulose (HPMC, AR), vinylidene fluoride (VDF, AR), hexafluoropropylene (HFP, AR), 2-(Trifluoromethyl) acrylic acid (MAF, AR), and diisopropyl peroxydicarbonate (IPP, AR) were obtained from Adamas (Shanghai, China). N, N-Dimethylacetamide (DMAc, 99%), sodium alginate (SA, CP), and sodium hydroxide (NaOH, 96%) were provided by Sinopharm Chemical Reagent Co., Ltd. (Shanghai, China). Bovine Serum Albumin (BSA, 98%) was purchased from Shanghai Yuanye Biotechnology Co., Ltd. (Shanghai, China) Polyethylene terephthalate (PET) nonwoven fabrics, used as the base membrane, were supplied by Teijin Limited (Tokyo, Japan). Unless otherwise specified, all reagents were used as received without further purification.

### 2.2. Synthesis and Characterization of PVHM Copolymers

**Figure 1:**
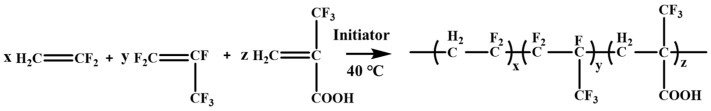
Synthesis process of amphiphilic copolymers.

PVHM copolymers (PVHMs) were synthesized via a radical suspension copolymerization process, as illustrated in Figure 1. First, deionized water and HPMC were added to a 5 L stainless steel autoclave and dissolved via stirring. After evacuation and purging with N_2_ to remove air, HFP, VDF, and MAF monomers were added to increase the reactor pressure to 2.0 MPa, followed by stirring at 500 rpm. Next, the reactor temperature was elevated to 40 °C, while continuing to add VDF from the high-pressure storage tank until the reactor pressure reached 6.0 MPa. The IPP initiator was then pumped into the reactor to initiate the polymerization reaction. Meanwhile, VDF was continuously added to maintain reactor pressure at 6.0 MPa until 1 kg VDF monomer, in total, was fully introduced, with simultaneous addition of IPP. The reaction temperature was maintained at 40 °C until the reactor pressure dropped to 3.0 MPa, at which point IPP addition ceased. When the reactor pressure dropped to 2.0 MPa, the polymerization reaction was concluded. Finally, the resulting polymer dispersion underwent degassing, water washing, filtration, and drying to yield the PVHM-1 copolymer. By altering the VDF/HFP/MAF monomer ratio and repeating the above steps, two PVHM copolymers with different carboxyl contents were obtained to prepare UF membranes.

The chemical composition of PVHMs was characterized by Fourier transform infrared spectroscopy via a KBr tablet (FTIR, TENSOR II., Ettlingen, Germany) and nuclear magnetic resonance spectroscopy in dimethyl sulfoxide-*d*_6_ (NMR, Bruker Avance III HD 500MHz, Rheinstetten, Germany). The number-average molecular weight (M_n_) and polydispersity index (PDI) of the PVHMs were characterized by gel permeation chromatography (GPC, Water-515, Milford, MA, USA) using N, N-dimethylformamide as the eluent.

### 2.3. Preparation and Characterization of PVHM Membranes

**Figure 2:**
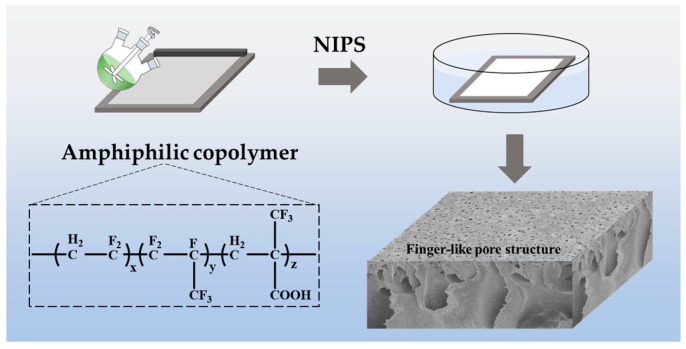
Schematic illustration of the fabrication procedure of UF membranes.

A typical NIPS method was adopted to prepare the PVHM membranes, as shown in Figure 2. First, PVHM powders were dissolved in DMAc solvent at 60 °C to yield homogeneous solutions with a concentration of 15 wt%. After degassing, the solution was cast on a non-woven substrate with a 250 μm scraper and then immersed into deionized water at 30 °C to form the solid membrane within 30 s. After being fully washed with water and dried at 60 °C, the resulting membranes were used for further characterization. To reveal the effect of carboxyl in PVHMs, pure PVDF membranes were also prepared and compared with PVHM membranes.

The surface composition of the membranes was characterized by attenuated total reflection Fourier transform infrared spectroscopy (ATR-FTIR, Nicolet 6700, Waltham, MA, USA) and X-ray photoelectron spectroscopy (XPS, PHI-5000, Eden Prairie, MN, USA). Static water contact angles were determined at 25 °C using a contact angle tester (OCA20, Data Physics Instruments GmbH, Filderstadt, Germany).

The surface zeta potentials of membranes were determined by flow potential measurements using a solid surface potential analyzer (Surge 3, Anton Paar GmbH, Graz, Austria). The measurements were conducted in a 1 mM KCl solution at 25 °C, with the pH adjusted via the addition of 0.01 M HCl or KOH. Zeta potential values were calculated using the Helmholtz–Smoluchowski equation [33].

The surface and cross-section morphologies of membranes were observed with a field emission scanning electron microscope (FE-SEM, SU8600, Hitachi, Tokyo, Japan). These membranes were cryogenically fractured in liquid nitrogen to preserve cross-section structure. The root-mean-square roughness (*Rms*) and surface morphology of the membrane were measured by atomic force microscopy (AFM, SPM Multimode, VEECO Instrumental, Plainview, NY, USA).

The pore sizes of the membranes were determined by a pore size analyzer (BSD-PB, Beijing, China). Pore size *D* was calculated via Equation (1) using perfluorotri-n-butylamine as the permeate.

(1) $$D=\frac{4\gamma \cos\theta }{p}$$

where γ is the surface tension of the liquid, θ is the contact angle, and p is the pressure differential.

Membrane porosity was determined by a gravimetric difference method. The membrane was cut into circular pieces with a diameter of 3.2 cm and then immersed in deionized water and weighed after the water on the surface was absorbed with paper. Then, the wet film was dried in a vacuum oven at 40 °C for 24 h to obtain its dry weight. The porosity (*ε*) was calculated using Equation (2).

(2) $$\varepsilon \%=\frac{W_{w}-W_{d}}{\rho_{w}Ad}\times 100$$

where $W_{w}$ (g) and $W_{d}$ (g) are the wet and dry film weights, respectively; $\rho_{w}$ is the density of the water (0.998 g/cm^3^); A (cm^2^) is the effective area of the membranes; and d (cm) is the thickness of the membranes.

### 2.4. Filtration Experiments

Filtration experiments were carried out on a cross-flow filtration (XFUX047, Millipore, Burlington, MA, USA) with an effective diameter of 56 mm at 1.0 bar, requiring the maintenance of a consistent flow rate to minimize experimental error. Prior to the test, all membranes were pre-pressurized at 1.5 bar for half an hour to achieve a steady state. In order to minimize measurement errors, three samples of each type of membrane were measured in parallel and averaged for the final results. The permeance *J_w_* (L·m^−2^·h^−1^·bar^−1^) was calculated using Equation (3).

(3) $$J_{w}=\frac{Q}{A\times ∆t\times ∆p}$$

where Q (L) is the permeate volume, A (m^2^) is the effective area of the membrane, ∆t (h) is the permeation time, and ∆p (bar) is the transmembrane pressure.

For BSA and SA retention tests, BSA, dissolved in a PBS buffer solution (1 g/L), and SA, dissolved in a 0.5 M KCl solution (100 mg/L), were used as feed solutions, respectively. A nanoparticle size potential analyzer (ZCEC, Malvern, UK) was used to test the size magnitude and electronegativity of BSA and SA in the feed solution. A UV-vis spectrometer (UV-1900i, Shimadzu, Kyoto, Japan) was used to determine the concentration of BSA in the feed and permeate solutions at 280 nm and the concentration of SA in the feed and permeate solutions at 200 nm. The retention (*R*) of BSA and SA was calculated using Equation (4).

(4) $$R\%=\left(1-\frac{C_{p}}{C_{f}}\right)\times 100$$

where $C_{p}$ and $C_{f}$ are the concentrations of the permeate and feed solutions, respectively.

### 2.5. Antifouling Test

The BSA static adsorption method was employed to characterize the membrane’s static antifouling property. The membrane operated as follows: a membrane sheet with a diameter of 10 mm was immersed in a 2 mL PBS solution for 30 min, resulting in the complete exchange of the water in the membrane sheet with the PBS solution. The membrane was then removed, and the residual PBS solution on the membrane surface was blotted out with dust-free paper. It was then placed into a 24-well plate with 2 mL of the BSA-PBS solution (1 g/L), and the plate was placed in a thermostatic oscillator with an oscillation rate of 100 rpm at 30 °C for 8 h to reach equilibrium. The concentration of BSA was determined using a UV-vis spectrometer (UV-1900i, Shimadzu, Kyoto, Japan) at 280 nm, and the amount of BSA adsorbed (A, µg cm^−2^) was calculated according to the following Equation (5).

(5) $$A=\left(C_{b}-C_{a}\right)\times \frac{V}{2S}$$

where $C_{b}$ is the BSA concentration before adsorption, $C_{a}$ is the BSA concentration after adsorption, V is the volume of the test liquid added, and S is the area of the membrane.

For the dynamic antifouling experiment, 1 g/L BSA-PBS was used as the feed solution in a cross-flow filtration device. The membrane was first pre-pressurized at 1.5 bar for 0.5 h; then, the filtration test was performed at 1.0 bar. Pure water permeance ($J_{w2}$) was measured for 0.5 h and recorded at 5 min intervals. Then, pure water was replaced with the BSA-PBS solution (1 g/L), and permeate permeance ($J_{p}$) was measured for 1 h and recorded at intervals of 5 min. Ultrasonication was performed for 10 min to remove contaminants adhering to the membrane surface. Finally, pure water permeance ($J_{w1}$) was measured again for 0.5 h.

In the dynamic antifouling experiment for SA polysaccharide, 10 mg/L and 100 mg/L SA-KCl solutions were used as the feed. Pure water permeance was measured over a period of 0.5 h, and pure water was replaced with a 10 mM KCl solution for filtration of 0.5 h. Then, the KCl solution was replaced with the SA-KCl solution, and permeate permeance was recorded over 1 h. After that, the membrane was ultrasonicated for 10 min to remove contaminant on the membrane surface. Finally, pure water permeance was measured again for 0.5 h. The effect of polymer composition on the antifouling property of the membranes was evaluated by calculating permeance recovery rate (FRR), the reversible contamination ratio ($R_{r}$), and the irreversible contamination ratio ($R_{ir}$) as follows.

(6) $$FRR\%=\frac{J_{w1}}{J_{w2}}\times 100$$

(7) $$R_{r}\%=\frac{J_{w1}-J_{p}}{J_{w2}}\times 100$$

(8) $$R_{ir}\%=\frac{J_{w2}-J_{w1}}{J_{w2}}\times 100$$

where $J_{w1}$ is pure water permeance after washing, $J_{w2}$ is initial pure water permeance, and $J_{p}$ is permeate permeance at the steady state.

### 2.6. Membrane Alkali-Resistant Study

PVDF, PVHM-1, and PVHM-2 membranes were immersed in a 0.1 M NaOH solution for 72 h at 25 °C for alkali treatment and then rinsed with deionized water to remove the residual NaOH solution. The alkali-treated membranes were named PVDF-B, PVHM-1B, and PVHM-2B. After air-drying, the membranes were characterized by ATR-FTIR and XPS to determine the chemical composition changes.

## 3. Results and Discussion

### 3.1. Characterization of PVHMs

**Figure 3:**
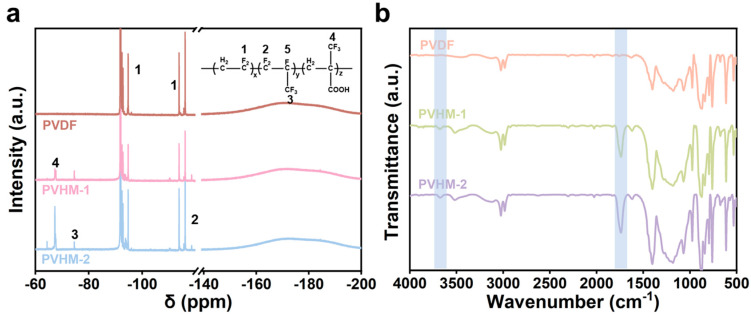
(**a**) ^19^F-NMR and (**b**) FTIR spectra of PVDF and PVHM copolymers.

Chemical composition and carboxyl groups of PVDF and PVHM polymers were analyzed using ^19^F-NMR spectroscopy and FTIR, respectively, as presented in Figure 3. Signals in the range of −91 ppm to −96 ppm corresponded to CH_2_CF_2_CH_2_CF_2_ derived from the head-to-tail linkage of VDF monomers, while signals observed between −113 ppm and −116 ppm corresponded to CH_2_CF_2_CF_2_CH_2_ originating from the tail-to-tail linkage of VDF monomers [34]. The characteristic peaks at −74.5 ppm, −118.6 ppm, and −184.3 ppm corresponded to the -CF_3_, -CF_2_, and -CF groups of the HFP monomer, respectively [35]. The peak at −67 ppm was assigned to the -CF_3_ group of the monomer MAF, and the enhanced intensity of this peak in PVHM-2 indicated a higher content of the MAF monomer compared to PVHM-1. Chemical composition was determined by integrating the areas of these characteristic peaks of each monomer, with the results summarized in Table 1. The increased weight ratio of MAF from 5.3% to 8.3% also meant more carboxyl in PVHM-2 than that in PVHM-1.

Comparing the infrared spectra of three polymers clearly revealed that PVHM copolymers exhibited two new absorption peaks, i.e., the C=O stretching vibration peak at 1734 cm^−1^ and the -OH stretching vibration peak at 3673 cm^−1^. Furthermore, the intensity of the C=O absorption peak gradually increased, indicating the successful incorporation of the hydrophilic component MAF into the polymer and an increase in the MAF content within the polymer, which is consistent with the results from the ^19^F-NMR tests.

The number-average molecular weight (Mn) and polydispersity index (PDI) of PVDF and PVHMs were characterized using GPC (Table 1). The Mn values of PVDF, PVHM-1, and PVHM-2 were 41.6 × 10^4^, 73.8 × 10^4^ Da, and 58.8 × 10^4^ Da, respectively, while PDI decreased from 2.5 to 1.9. Generally, molecular weight and its distribution of polymers significantly influenced the mechanical properties and membrane structure. Polymeric membranes derived from polymers with higher molecular weight and lower PDI exhibit superior mechanical properties. The Mn of synthesized PVHMs was higher than that of commercial PVDF for ultrafiltration membrane production, suggesting that PVHMs could meet the mechanical strength requirements for real application.

From these results, it can be seen that the proposed method should contribute an efficient carboxylated PVDF copolymer candidate for improved hydrophilic membranes, which will be described in the following sections.

### 3.2. Composition, Hydrophilicity, and Charge State of PVHM Membranes

**Figure 4:**
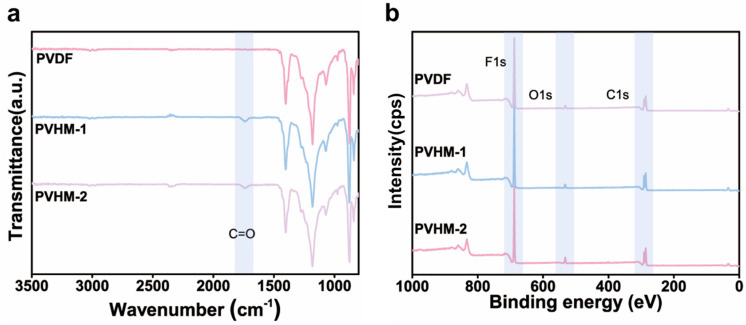
(**a**) ATR-FTIR and (**b**) XPS spectra of PVDF and PVHM membranes.

The chemical compositions of the surface layers of the PVHM membranes were characterized using ATR-FTIR and XPS (Figure 4). As shown in Figure 4a, distinct stretching vibration peaks of C=O at 1736 cm^−1^ emerged for both PVHM-1 and PVHM-2 membranes, in contrast to PVDF membranes. This observation indicates the presence of the MAF component in the membrane surface layer.

XPS spectra (Figure 4b) revealed the characteristic peaks of C1s (286.2 eV), O1s (532.8 eV), and F1s (688.1 eV) for PVDF and PVHM membranes [19]. The intensity of the O1s peak on the PVHM membrane surface increased with the MAF content in the copolymer, as shown in Table 2. Compared with the synthesized PVHMs, a higher MAF content in surface layer was found, indicating the enrichment of the hydrophilic MAF in membranes. This enrichment was generated from a typical surface segregation mechanism [28,36,37] in which the hydrophilic MAF units exhibited stronger interaction with a water coagulation bath during the membrane formation. The preferential migration of MAF segments towards the interface between the casting solution and the coagulation bath, i.e., the membrane surface and internal-pore-wall surface, occurred. It should be noted that the random distribution and small size of the MAF segments in PVHMs limited their migration; thus, the surface enrichment ratio was lower than for the hydrophilic components in block, comb-like, or hyperbranched amphiphilic copolymers [38]. In aiming towards large-scale production and application, however, the PVHMs in this work should be the more acceptable amphiphilic copolymers for UF membranes.

**Figure 5:**
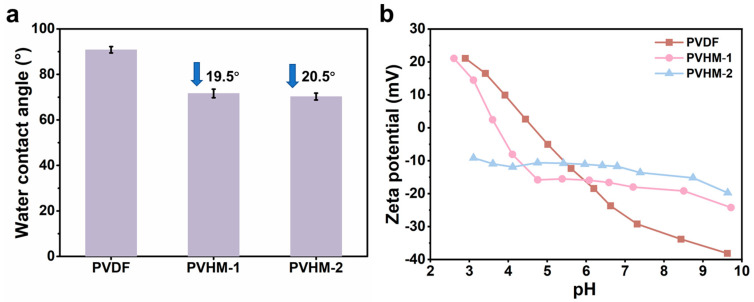
(**a**) Water contact angles and (**b**) surface zeta potentials of PVDF and PVHM membranes. The arrow indicated a decrease in the water contact angle compared to PVDF membranes.

**Figure 6:**
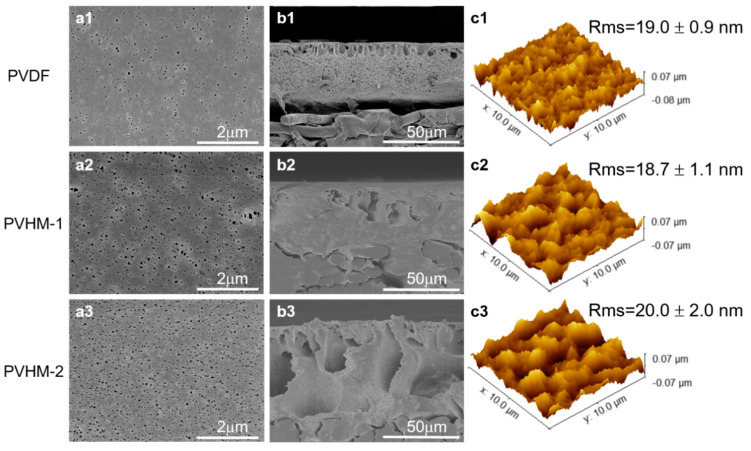
SEM images of (**a1**–**a3**) membrane surface and (**b1**–**b3**) cross-section; (**c1**–**c3**) AFM images of PVDF and PVHM membranes.

Hydrophilicity plays a crucial role in membrane permeability and fouling resistance. It is influenced by two factors: (1) the nature of the material, and (2) surface roughness. In this study, the water contact angles of PVHM membranes were smaller than those of PVDF membranes, as shown in Figure 5a. This was primarily due to the hydrophilic carboxyl component MAF in PVHMs, rather than surface roughness, as demonstrated by the minor roughness variations shown in Figure 6. The higher the MAF content, the better the hydrophilicity of the corresponding membranes.

The zeta potentials of membranes at various pH levels are shown in Figure 5b. When carboxyl was incorporated, the membrane’s isoelectric point (IP) decreased. The IPs of the PVDF and PVHM-1 membranes were 4.7 and 3.6, respectively, indicating the function of carboxyl. Notably, PVHM-2 membranes did not exhibit an isoelectric point over a wide pH range, suggesting a negative property generated from the higher carboxyl units. These characteristics of the PVHM membranes imply that the corresponding membrane should respond with improved rejection and antifouling to the negatively charged substance.

### 3.3. Morphologies of PVHM Membranes

Surface and cross-section morphologies of UF membranes were observed by SEM and AFM (Figure 6). Fewer pores appeared on the surface of PVDF membranes, and cross-sectional views revealed shorter finger-like pores. As the hydrophilic MAF content increased, the larger finger-like pores gradually extended through the entire cross-section of the PVHM membrane. These changes in the membrane pores were typical of amphiphilic copolymers in the NIPS process, which is attributed to the stronger interaction between the hydrophilic component and water in the coagulation bath. During phase inversion, in the mechanism, the exchange rate between the solvent DMAc and the non-solvent water accelerates, leading to instantaneous phase separation and the formation of large finger-like pores [39]. In addition, AFM results show that the PVDF and PVHM membranes exhibit similar surface roughness, indicating that carboxylated PVDF has a negligible effect on membrane surface roughness.

### 3.4. Pore Size and Porosity PVHM Membranes

**Figure 7:**
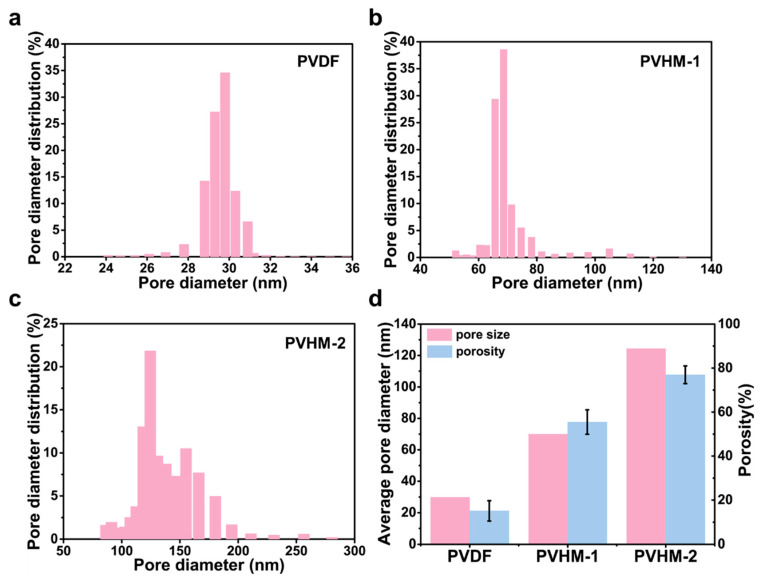
(**a**–**c**) Pore diameter distribution. (**d**) Average pore size and porosity of PVDF and PVHM membranes.

Pore diameter and distribution and average porosity, determined using the bubble and dry-wet weight methods [40,41], are given in Figure 7. The average pore sizes of the PVDF, PVHM-1, and PVHM-2 membranes were 29.9 nm, 70 nm, and 124.5 nm, respectively, while the porosities of the PVDF, PVHM-1, and PVHM-2 membranes were 15.1%, 55.5%, and 76.9%, respectively. Based on the fact that membrane structure depends on the mutual diffusion rate between the solvent and the coagulation bath, the presence of hydrophilic segments in PVHMs accelerates the rate of water penetration into the membrane during membrane formation. Thereby, macro pores and voids are formed within the final PVHM membranes.

### 3.5. Separation Performance of PVHM Membranes

**Figure 8:**
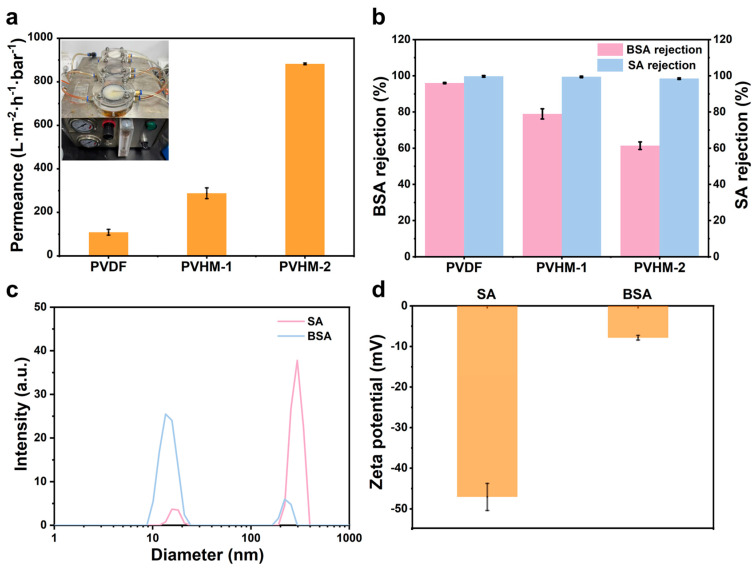
(**a**) Pure water permeance. The inset is a physical diagram of the cross-flow filtration apparatus for filtration experiments. (**b**) Rejection of BSA and SA for PVDF and PVHM membranes. (**c**) Particle size and (**d**) zeta potential of BSA and SA solutes in feeds.

To investigate the influence of polymer chemical composition on membrane permeation and separation performance, pure water permeance and the rejection of negatively charged BSA proteins and SA polysaccharides as model solutes were tested for the prepared PVDF and PVHM membranes (Figure 8). The pure water permeance of PVHM membranes significantly increased in comparison with PVDF membranes (Figure 8a). This result was not only consistent with the increasing membrane pore size and porosity, as mentioned above; the carboxyl unit enabled the PVHM membranes with enhanced hydrophilicity to reduce water permeation resistance.

The rejection of negatively charged BSA and SA solutes is shown in Figure 8b. The retention rate of BSA decreased from 96.0% for PVDF membranes to 61.4% for PVHM-2 membranes. Considering that the size of most of the BSA aggregation particles was 11.5 nm in the feeds (Figure 8c), the lower retention rate of the PVHM membranes for BSA was mainly attributed to both the larger pore size of the PVHM membranes and the smaller size of the BSA aggregation particles. In comparison, the retention rate of SA exceeded 99% for two PVHM membranes. This excellent separation performance originated not only from the larger size of the SA aggregation particles in the feeds (e.g., 145.6 nm) (Figure 8c) but also from the electrostatic repulsion between SA (e.g., −47 mV) (Figure 8d) and the membranes (Figure 5b).

### 3.6. Fouling Resistance of PVHM Membranes

**Figure 9:**
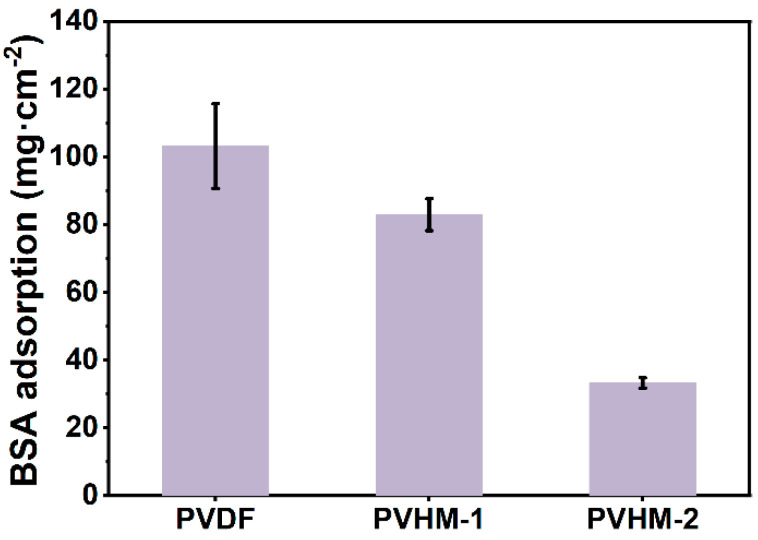
BSA adsorption on PVDF and PVHM membranes.

The static adsorption of BSA on the membrane surface is presented in Figure 9. Compared with the adsorption content of 103.2 μg·cm^−2^ on pure PVDF membranes, the BSA absorption content was 82.9 μg·cm^−2^ and 33.2 μg·cm^−2^ on PVHM-1 and PVHM-2 membranes, respectively. These data clearly indicate that the carboxyl unit efficiently reduced BSA absorption and fouling tendency due to the intrinsically negative repulsion between BSA and the PVHM membranes, as well as improved membrane hydrophilicity.

**Figure 10:**
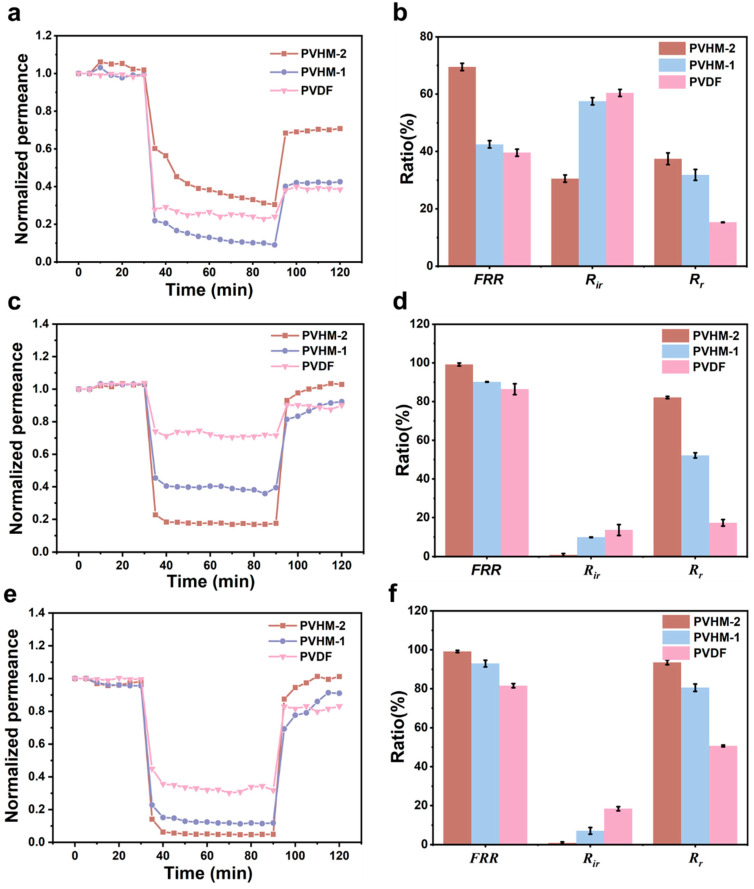
Normalized permeance changes with elapsed time and fouling resistance during the filtration of (**a**,**d**) BSA solution, (**b**,**e**) 10 mg/L SA, and (**c**,**f**) 100 mg/L SA.

The dynamic anti-fouling property of PVHM membranes was further evaluated by filtering BSA and SA as model contaminants. As shown in Figure 10, two carboxylated PVDF membranes demonstrated better antifouling and permeance recovery properties than pure PVDF membranes. Based on the well-known fouling mechanism [42], the PVDF membranes’ stronger hydrophobicity and weaker negative repulsion to BSA indicated an obvious BSA fouling tendency. In comparison, higher hydrophilicity and stronger negative repulsion to BSA contributed to improved BSA fouling resistance on PVHM membranes.

To exclude the influence of membrane pore size and solute particle size on antifouling properties [43], filtration-wash experiments involving SA solute particles of 145.6 nm using PVHM membranes (i.e., PVHM-2 with a pore size of 124.5 nm) were carried out to better demonstrate the antifouling properties of PVHM membranes. As can be seen in Figure 10, two PVHM membranes demonstrated greatly increased fouling resistance to SA when compared with pure PVDF membranes. For PVHM-2 membranes, *FRR* and *Rr* achieved results as high as 99.1% and 93.6% when used for the filtration of 100 mg/L SA solutions, whereas the *FRR* and *Rr* of the pure PVDF membranes reached as low as 81.6% and 50.6%, respectively. These results confirm that incorporating carboxyl into PVDF can efficiently improve the antifouling performance, which essentially depends on the PVHM membranes’ hydrophilicity and the negative charge repulsion between the membranes and the SA solutes.

### 3.7. Alkali Resistance of PVHM Membranes

**Figure 11:**
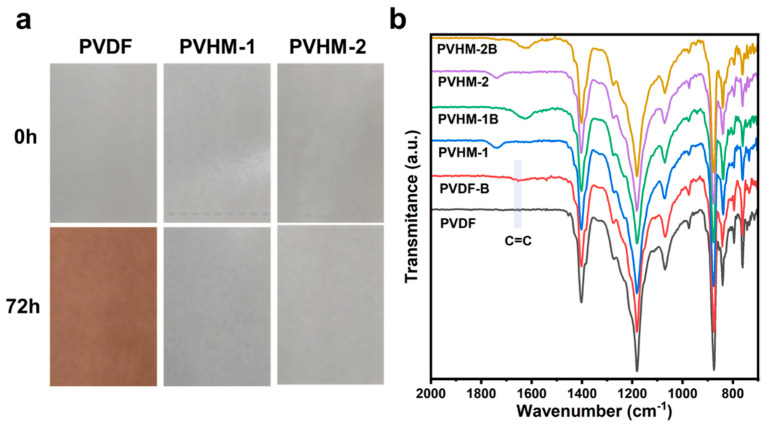
(**a**) Digital photos and (**b**) ATR-FTIR spectra of PVDF and PVHM membranes before and after alkali treatment.

Figure 11a shows digital photos of the PVDF and PVHM membranes before and after immersion in a 0.1 M NaOH aqueous solution for 72 h. The PVDF membrane turned an obvious brown color in the alkali solution, indicating a chemical reaction between the alkali and pure PVDF. On the contrary, the PVHM membranes’ color remained almost unchanged, suggesting a greater resistance to alkali. Based on the elimination of HF and the formation of C=C bonds for the PVDF degradation mechanism, further information was extracted from the membrane composition changes before and after alkali treatment. From the ATR-FTIR spectra (Figure 11b), peaks at 1640–1680 cm^−1^ were found for the treated PVDF membranes, implying C=C bond formation as a result of the elimination of HF. For PVHM membranes, the absence of C=C bonds suggests that the elimination of HF was suppressed to certain degree. The disappearance of peaks at 1737 cm^−1^ for C=O units in -COOH and the appearance of peaks at 1625 cm^−1^ for -COO^−^Na^+^ indicates a change in the -COOH groups in the NaOH solution. As to the mechanism of action, it could have been the electron-rich nature of the negative -COOH and -COO^−^Na^+^ groups that effectively decreased the attacking activity of the OH^−^ anion, thus restraining the elimination of HF.

Though it is difficult to reveal more precisely, the surface chemical composition according to the XPS data (Table 3) indicates an obvious decrease in the F/C ratio for the pure PVDF membranes and the PVHM-1 membranes containing less -COOH after treatment with NaOH. For the PVHM-2 membranes containing more -COOH, the very low F/C ratio reduction (from 81.1% to 79.3%) indicates its excellent alkali resistance. Importantly, the improved alkali resistance of PVHM could contribute additional advantages of PVDF-based membranes in industrial practices.

## 4. Conclusions

A new type of amphiphilic copolymer, carboxylated PVDF, was proposed and successfully synthesized via a facile free-radical copolymerization process by adopting carboxyl-containing monomers. By investigating the corresponding PVHM membranes formed via an NIPS route, we found that the PVHM containing 8.3 wt% MAF could efficiently endow the membranes with strong hydrophilicity and a stable, negatively charged surface, thus enabling the membranes to exhibit low BSA absorption, excellent fouling resistance, and retention with respect to negatively charged solutes and foulants. Taking into account a number of additional advantages, e.g., the easy mass-production, high molecular weight, and good alkali resistance of PVHMs, such carboxylated PVDF membranes have obvious potential in the industrial production and application of high-performance UF membranes, and further investigation into their scaled-up production and comprehensive performance is warranted.

## Figures and Tables

**Table 1:** Chemical composition and molecular weight of PVDF and PVHM copolymers.

Copolymer	VDF (wt%)	HFP (wt%)	MAF (wt%)	M_n_ (g/mol)	PDI
PVDF	100.0	-	-	41.6 × 10^4^	2.5
PVHM-1	93.6	1.1	5.3	73.8 × 10^4^	2.2
PVHM-2	91.2	0.5	8.3	58.8 × 10^4^	1.9

**Table 2:** Surface chemical composition of PVHM membranes.

Membrane	Atomic Concentration (at%)			MAF * (wt%)
	C	F	O	
PVDF	52.16	44.92	2.91	-
PVHM-1	49.94	46.89	3.17	14.40
PVHM-2	52.56	42.63	4.81	22.19

* calculated from atom ratios of C, F, and O.

**Table 3:** Chemical composition of PVDF and PVHM membranes before and after alkali treatment.

Membrane	Atomic Concentration (at%)				F/C (at%)
	C	F	O	Na	
PVDF	52.16	44.92	2.91	-	86.1
PVHM-1	49.94	46.89	3.17	-	93.9
PVHM-2	52.56	42.63	4.81	-	81.1
PVDF-B	60.89	32.89	5.91	0.32	54.0
PVHM-1B	56.87	36.97	5.30	0.87	65.0
PVHM-2B	52.46	41.62	4.44	1.47	79.3

## Data Availability

The original contributions presented in this study are included in the article. Further inquiries can be directed to the corresponding author.

## References

[1] Nguyen T.A., Juang R.-S. (2013). Treatment of waters and wastewaters containing sulfur dyes: A review. Chem. Eng. J..

[2] Dasgupta J., Sikder J., Chakraborty S., Curcio S., Drioli E. (2015). Remediation of textile effluents by membrane based treatment techniques: A state of the art review. J. Environ. Manag..

[3] Thamaraiselvan C., Noel M. (2015). Membrane processes for dye wastewater treatment: Recent progress in fouling control. Crit. Rev. Environ. Sci. Technol..

[4] Chen X., Zhao Y., Moutinho J., Shao J., Zydney A.L., He Y. (2015). Recovery of small dye molecules from aqueous solutions using charged ultrafiltration membranes. J. Hazard. Mater..

[5] Liu F., Hashim N.A., Liu Y., Abed M.R.M., Li K. (2011). Progress in the production and modification of PVDF membranes. J. Membr. Sci..

[6] Chen L., Si Y., Zhu H., Jiang T., Guo Z. (2016). A study on the fabrication of porous PVDF membranes by in-situ elimination and their applications in separating oil/water mixtures and nano-emulsions. J. Membr. Sci..

[7] Almaie S., Vatanpour V., Rasoulifard M.H., Seyed Dorraji M.S. (2022). Novel negatively-charged amphiphilic copolymers of PVDF-g-PAMPS and PVDF-g-PAA to improve permeability and fouling resistance of PVDF UF membrane. React. Funct. Polym..

[8] Ahmad N.N.R., Mohammad A.W., Mahmoudi E., Ang W.L., Leo C.P., Teow Y.H. (2022). An overview of the modification strategies in developing antifouling nanofiltration membranes. Membranes.

[9] Sun T., Zhu Z., Qiu Z., Yang W., Jiang Y., Su Q., Zhu L., Zhao Z., Huang X., Xue Y. (2025). Preparation of hybrid nanofiltration membranes based on Schiff base reaction for hard water softening. J. Membr. Sci..

[10] Cheng Q., Zhao Z., Zhu Z., Zhang S., Wang L., Wang C., Cao Z., Pan L., Zhu L., Zhu B. (2025). Preparation and properties of acrylonitrile-DMAEMA copolymer based crosslinked and positively-charged nanofiltration membranes. J. Membr. Sci..

[11] Cui R., Hao K., Xue Y., Wang C., Shen S., Zhao Z., Zhao Y., Ling J., Zhu B., Fang L. (2024). A novel amphiphilic polypeptoid based ultrafiltration membrane with excellent biocompatible property. J. Membr. Sci..

[12] Ma W., Rajabzadeh S., Shaikh A.R., Kakihana Y., Sun Y., Matsuyama H. (2016). Effect of type of poly(ethylene glycol) (PEG) based amphiphilic copolymer on antifouling properties of copolymer/poly(vinylidene fluoride) (PVDF) blend membranes. J. Membr. Sci..

[13] Luo T., Lin S., Xie R., Ju X.-J., Liu Z., Wang W., Mou C.-L., Zhao C., Chen Q., Chu L.Y. (2014). pH-responsive poly(ether sulfone) composite membranes blended with amphiphilic polystyrene-block-poly(acrylic acid) copolymers. J. Membr. Sci..

[14] Asatekin A., Kang S., Elimelech M., Mayes A.M. (2007). Anti-fouling ultrafiltration membranes containing polyacrylonitrile-graft-poly(ethylene oxide) comb copolymer additives. J. Membr. Sci..

[15] Zhu M.M., Fang Y., Chen Y.C., Lei Y.Q., Fang L.F., Zhu B.K., Matsuyama H. (2021). Antifouling and antibacterial behavior of membranes containing quaternary ammonium and zwitterionic polymers. J. Colloid Interface Sci..

[16] Zhou Z., Rajabzadeh S., Rajjak Shaikh A., Kakihana Y., Ishigami T., Sano R., Matsuyama H. (2016). Preparation and characterization of antifouling poly(vinyl chloride-co-poly(ethylene glycol)methyl ether methacrylate) membranes. J. Membr. Sci..

[17] Wang S.Y., Fang L.F., Cheng L., Jeon S., Kato N., Matsuyama H. (2018). Novel ultrafiltration membranes with excellent antifouling properties and chlorine resistance using a poly(vinyl chloride)-based copolymer. J. Membr. Sci..

[18] Seo J.Y., Choi Y.J., Kang Y., Baek K.Y. (2024). Protection group assisted non-solvent induced phase separation for PO3H2 functionalized PVDF ultrafiltration membranes with a heavy metal removal capability. J. Membr. Sci..

[19] Zhang L., Zhu Z., Azhar U., Ma J., Zhang Y., Zong C., Zhang S. (2018). Synthesis of well-defined PVDF-based amphiphilic block copolymer via iodine transfer polymerization for antifouling membrane application. Ind. Eng. Chem. Res..

[20] Zhang X., Liang Y., Ni C., Li Y. (2021). Anti-biofouling microfiltration membranes based on 1-vinyl-3-butylimidazolium chloride grafted PVDF with improved bactericidal properties and vitro biocompatibility. Mater. Sci. Eng. C-Mater. Biol. Appl..

[21] Liu L., Huang L., Shi M., Li W., Xing W. (2019). Amphiphilic PVDF-g-PDMAPMA ultrafiltration membrane with enhanced hydrophilicity and antifouling properties. J. Appl. Polym. Sci..

[22] Rahimpour A., Madaeni S.S., Ghorbani S., Shockravi A., Mansourpanah Y. (2010). The influence of sulfonated polyethersulfone (SPES) on surface nano-morphology and performance of polyethersulfone (PES) membrane. Appl. Surf. Sci..

[23] Hu M., Cui Z., Yang S., Li J., Shi W., Zhang W., Matindi C., He B., Fang K., Li J. (2021). Pregelation of sulfonated polysulfone and water for tailoring the morphology and properties of polyethersulfone ultrafiltration membranes for dye/salt selective separation. J. Membr. Sci..

[24] Li S., Cui Z., Zhang L., He B., Li J. (2016). The effect of sulfonated polysulfone on the compatibility and structure of polyethersulfone-based blend membranes. J. Membr. Sci..

[25] Liu Y., Yue X., Zhang S., Ren J., Yang L., Wang Q., Wang G. (2012). Synthesis of sulfonated polyphenylsulfone as candidates for antifouling ultrafiltration membrane. Sep. Purif. Technol..

[26] Wang N., Wang J., Zhang P., Wang W., Sun C., Xiao L., Chen C., Zhao B., Kong Q., Zhu B. (2017). Metal cation removal by P(VC-r-AA) copolymer ultrafiltration membranes. Front. Chem. Sci. Eng..

[27] Wang N.C., Fang L.F., Wang J., Zhang P., Wang W.B., Lin C.E., Xiao L., Chen C., Zhao B., Abdallah H. (2018). pH-dependent property of carboxyl-based ultrafiltration membranes fabricated from poly(vinyl chloride-r-acrylic acid). J. Appl. Polym. Sci..

[28] Hester J.F., Banerjee P., Won Y.Y., Akthakul A., Acar M.H., Mayes A.M. (2002). ATRP of amphiphilic graft copolymers based on PVDF and their use as membrane additives. Macromolecules.

[29] Hashim N.A., Liu F., Li K. (2009). A simplified method for preparation of hydrophilic PVDF membranes from an amphiphilic graft copolymer. J. Membr. Sci..

[30] Chen J., Meng X., Tian Y., Wang X., Zhu J., Zheng H., Wang L. (2020). Fabrication of a superhydrophilic PVDF-g-PAA@FeOOH ultrafiltration membrane with visible light photo-fenton self-cleaning performance. J. Membr. Sci..

[31] Meyer J., Ulbricht M. (2018). Poly(ethylene oxide)-block-poly(methyl methacrylate) diblock copolymers as functional additive for poly(vinylidene fluoride) ultrafiltration membranes with tailored separation performance. J. Membr. Sci..

[32] Wang J., Li C., Han K., Yuan J., Pan Z., Pan M. (2023). Synthesis of amphiphilic functional terpolymers towards preparation of high flux, anti-fouling, micropollutant-capturing ultrafiltration membranes. J. Clean. Prod..

[33] Möckel D., Staude E., Dal-Cin M., Darcovich K., Guiver M. (1998). Tangential flow streaming potential measurements: Hydrodynamic cell characterization and zeta potentials of carboxylated polysulfone membranes. J. Membr. Sci..

[34] Katoh E., Ogura K., Ando I. (1994). An NMR study of poly(vinylidene fluoride) structure by ^1^H, ^13^C, and ^19^F triple resonance method. Polym. J..

[35] Twum E.B., McCord E.F., Fox P.A., Lyons D.F., Rinaldi P.L. (2013). Characterization of backbone structures in poly(vinylidene fluoride-co-hexafluoropropylene) copolymers by multidimensional ^19^F NMR spectroscopy. Macromolecules.

[36] Hester J.F., Banerjee P., Mayes A.M. (1999). Preparation of protein-resistant surfaces on poly(vinylidene fluoride) membranes via surface segregation. Macromolecules.

[37] Hester J.F., Mayes A.M. (2002). Design and performance of foul-resistant poly(vinylidene fluoride) membranes prepared in a single-step by surface segregation. J. Membr. Sci..

[38] Yi Z., Zhu L., Xiong R., Fang C., Zhu B., Zhu L., Zeng H. (2024). Advanced functional membranes based on amphiphilic copolymers. Prog. Polym. Sci..

[39] Shen J., Zhang Q., Yin Q., Cui Z., Li W., Xing W. (2017). Fabrication and characterization of amphiphilic PVDF copolymer ultrafiltration membrane with high anti-fouling property. J. Membr. Sci..

[40] Shi F., Ma Y., Ma J., Wang P., Sun W. (2012). Preparation and characterization of PVDF/TiO_2_ hybrid membranes with different dosage of nano-TiO_2_. J. Membr. Sci..

[41] Zhang G., Lu S., Zhang L., Meng Q., Shen C., Zhang J. (2013). Novel polysulfone hybrid ultrafiltration membrane prepared with TiO_2_-g-HEMA and its antifouling characteristics. J. Membr. Sci..

[42] Zhang C., Bao Q., Wu H., Shao M., Wang X., Xu Q. (2022). Impact of polysaccharide and protein interactions on membrane fouling: Particle deposition and layer formation. Chemosphere.

[43] Wang Y., Zheng X., Li D., Meng F., Tian J., Wang M., Li L., Wu H., Zhang Y. (2022). Effect of sodium and potassium on polysaccharide fouling on PVDF and graphene oxide modified PVDF membrane surfaces. Process Saf. Environ..

